# The efficacy of online extensive reading among university students and the relationship between affective variables and english reading comprehension

**DOI:** 10.1038/s41598-025-92326-9

**Published:** 2025-03-11

**Authors:** Helta Anggia, Anita Habók

**Affiliations:** 1 Doctoral School of Education, University of Szeged, Szeged, Hungary; 2 Institute of Education, Digital Learning Technologies Incubation Research Group, University of Szeged, MTA-SZTE Digital Learning Technologies Research Group, Szeged, Hungary

**Keywords:** Statistics, Psychology and behaviour, Mathematics and computing

## Abstract

Online extensive reading has been shown to improve English reading comprehension and related motivational factors. However, few studies have examined the structural relationship between affective variables and reading comprehension in this platform. Thus, this study designed an online extensive reading program to examine this relationship and the affective behavior of a sample of university students. Clustered random sampling was used to select two experimental groups (N = 120, N = 130) and a control group (N = 100), who were requested to take the Test of English as a Foreign Language reading comprehension pre-and post-tests and complete online surveys about self-beliefs, reading motivation, and English reading behavior. Based on the findings, the experimental groups significantly outperformed the control group in reading comprehension. Meanwhile, reading self-efficacy predicted intrinsic and extrinsic motivation, but self-concept only predicted extrinsic drive. Although there was no correlation between extrinsic or intrinsic motivation and English reading comprehension, only the latter mediated self-efficacy with English reading behavior. Surprisingly, reading behavior did not mediate intrinsic motivation and reading comprehension. The findings imply that internal motivation and reading behavior cannot guarantee students’ reading comprehension achievement.

## Introduction

According to self-related beliefs theory, students with academic self-concept (SC) tend to excel in reading motivation^1,2^. However, many studies have also suggested that academic SC is a strong predictor of reading achievement^3,4^, with reading self-efficacy (RSE) having a positive impact on reading motivation^3^ and English reading comprehension (ERC)^5^. In related research, Mikami^6^ revealed an association between intrinsic motivation (IM) and positive reading achievement, while Sani et al.^4^ found that extrinsic motivation (EM) positively influences ERC, especially during examinations. Additionally, reading habits have been shown to promote English reading ability and the achievement of reading goals^7,8^. Based on these findings, many scholars have concurred that self-related beliefs, reading motivation, and English reading behavior (ERB) play important roles in ERC achievement. Researchers have also conducted experimental studies regarding the positive impact of online extensive reading (ER) on ERC and related motivational factors. However, few studies have examined the structural or complex relationship between affective variables such as self-concept, reading self-efficacy, intrinsic motivation, extrinsic motivation, English reading behavior, and English reading comprehension in online extensive reading platforms. This study’s objective was to implement an online ER program, to compare the effect of using online ER platforms and offline ER activity on students’ English reading achievement, and to examine the relationship between students’ English reading comprehension and the aforementioned affective behaviors of a sample of university students. This study contributed to the field of reading by showcasing that self-related beliefs, reading motivation, and English reading behavior are important aspects that teachers need to consider and work upon when teaching English reading comprehension. The result of this study added new insights towards Extensive Reading studies that have rarely discussed the structural relationship between self-related beliefs, reading motivation, English reading behavior and English reading comprehension. Gaining a more thorough understanding of these structural relationships is crucial for designing holistic interventions that address multiple emotional factors at once, aiming to enhance reading comprehension outcomes.

## Conceptual framework

### Comprehensible input theory in second language acquisition

Patrick^9^ affirmed the application of Krashen’s principle of comprehensible input in L2 instruction. He delivered an experience-based confirmation that the language that is acquired lasts longer than the one that is learned. The components of Krasehn’s comprehensible input theory are the acquisition-learning distinction principle, the natural order principle, the monitor principle, the input principle, the affective filter principle, and the compelling principle^10^.

Patrick^9^ elaborated the principles of comprehensible input theory. First, *the acquisition-learning distinction principle* pertains to language learning situations in which learners do not acquire all of the learned language components. This principle asserts that language instructors should place a greater emphasis on acquisition than on learning methods. Students can acquire L2 more effectively if they embrace messages that are easily understood. Second, *the natural order principle* asserts that language is acquired in its natural order. No matter how qualified the instructor is or how effective the learning method is, students will only acquire the language that corresponds to their current situation and needs. Therefore, teachers must bear in mind that not all taught L2 skills can be mastered and acquired by students at the expected time. When the time is perfect, they will acquire the language. Third, *the monitor principle*, on the other hand, asserts that language learners are capable of monitoring every utterance they create. Typically, learners’ grammatical skills perform this function when editing their language production. However, this aptitude should not interfere too much with the production of language. Otherwise, it will obscure linguistic precision. Meanwhile, the fourth principle, *the input principle* holds that L2 learning output (speaking and writing) must be the direct results of the input (listening and reading). Language acquisition occurs through listening and reading. Listening and reading are two language abilities that can be explored for the acquisition of a language by students. The fifth principle, *the affective filter principle* contains the emotive aspect of second language acquisition. We cannot deny that emotions and feelings can both facilitate and impede language acquisition. Reducing tension during language acquisition can reduce anxiety. Explicit learning typically induces extreme anxiety. Last but not least, according to the sixth principle, *compelling principle*, it is simpler for a person to learn a language if the materials studied are compelling and interesting. An extensive reading program, for instance, allows readers to autonomously select whatever books or texts most pique their interest. As language acquisition is an unconscious process, compelling materials are useful for helping pupils acquire the target language unconsciously.

Therefore, the aim of this study was to compare the effect of the compelling and the non-compelling online extensive reading media on the students English reading comprehension achievement. This investigation is crucial to the realization of the comprehensible input theory. Students are afforded the opportunity to acquire language naturally through online extensive reading. The language in an extensive reading program is presented in the form of comprehensible input, so it is also engaging for students because they can select reading material that piques their interest. Extensive reading is also a program that is conducted without the explicit purpose of teaching the language, allowing students to avoid anxiety when learning the language. During their reading activity in the online extensive reading program, students can also apply their prior grammatical knowledge and enhance their understanding, particularly in terms of language production as an output of their reading.

### Online extensive reading

A review of 15 studies has characterized online extensive reading based on its role in reading learning and its effect on reading achievement, and reading motivation. Online extensive reading has two main features that are useful for reading, namely the hypermedia and the learning management system facility. Students who utilized online learning materials for ER rather than paper-based ER approaches reported greater comprehension of a variety of topics^11^. For instance, students could refer to Wikipedia, YouTube, and many other media to dig for more information about specific subjects in their reading. In some instances, they could also use interactive eBook features to emphasize, annotate, summarize stories, and maintain essential glossaries. Thus, multimodality was essential for enhancing students’ comprehension of English texts^12–15^. Rezaee and Farahian^16^ found evidence to support the multimodality argument by demonstrating that online ER activities helped students improve their reading achievement. Using a screen, students engaged in a substantial amount of reading activity. The websites’ hypermedia aided the students’ comprehension of the reading material since they could use other information resources to gain full comprehension.

In the meantime, existing research has also demonstrated that a learning management system fosters learning autonomy in students^17^. Xreading, for example, is the most frequently utilized ER website in the studies reviewed^18–20^. This website provides a broad variety of customized reading material with varying levels of difficulty. The Xreading system enables students to read a text or book to accomplish a predetermined number of word requirements set by the teacher. This system tracks the texts that students retrieve and return. When students complete tasks that include reading a book, answering relevant quizzes on the content, and returning the book, the system records their reading achievement and allows instructors to provide feedback based on the students’ reading records. After the reading task, students can share information about the materials they have read with their peers and teacher. In doing so, online ER incorporates at least two key principles of Keller’s instructional model: control and satisfaction^21^. Reading management systems provide students with a sense of control over the content they read and a degree of gratification with their skill development. Some of the selected studies also employed Mreader, Moodle Reader, and Goodreads^22–26^. In a few instances, the authors also used websites that they had created themselves for online ER programs. These websites were useful and encouraging to the students. Students can be encouraged to read more if internet-based reading management systems and reading resources are utilized in conjunction with online ER.

### Related affective aspects

Existing empirical studies have indicated that reading motivation has a significant impact on academic achievement, including ERC. For example, Marsh et al.^27^ and Miyamoto et al.^22^ found that reading motivation is a significant predictor of ERC, while Wigfield and Eccles^13^ suggested that academic SC can affect the value and expectation components of such motivation. Conversely, academic SC has a positive and statistically significant relationship with academic achievement, through the mediation of IM^18,19^. In other words, academic SC plays a crucial role in determining reading motivation, which, in turn, influences ERC.

Meanwhile, Schunk and DiBenedetto^17^ argued that self-efficacy beliefs are essential for motivating students to engage in reading and overcoming related obstacles. In addition, Schöber et al.^28^ found evidence of reciprocal effects between self-efficacy and achievement, based on the skill development model. At the same time, a number of research proposed that self-concept is a strong predictor of reading achievement^29–32^. In related research, Jiang^18^, Luther^19^, and Yang et al.^3^ emphasized the significance of emotional factors when investigating the relationship between self-efficacy in reading a foreign language and reading performance. As for IM, Luther^19^ and Yang et al.^3^ found that it mediates self-efficacy with reading behavior. In sum, self-efficacy can influence ERC and behavior through the mediation of motivation.

Finally, the development of reading comprehension ability is associated with both EM and IM. In this regard, Sani et al.^4^ found that students are more extrinsically motivated to accomplish reading assignments and attain the desired reading score. Conversely, Miyamoto et al.^22^ and Wang et al.^23^ discovered significant indirect effects of IM on ERC via reading volume and metacognitive understanding of strategy use. Reading behavior has also been found to have a direct positive influence on ERC^27^, with some empirical studies finding that positive reading habits can be instilled by using ER^15,28,33^. Regarding the relationship between ERC and ERB, several studies have found a relationship between reading and the pleasure that comes from reading. Beglar et al. (2012)^34^ examined the extent to which reading for pleasure affected participants’ reading performance. They also investigated how the reading comprehension of the pleasure reading group developed during the programme. In their research, they found from student feedback that students who read for pleasure were more motivated and encouraged to read. The role of reading pleasure was also confirmed by Arnold (2009)^35^. She investigated the linguistic and affective aspects of the ER programme and how it benefited learners. She also examined students’ engagement in reading, whether they were willing to read out-of-classroom. The results showed that the ER programme was a pleasure to read and motivating for the participants. Their confidence increased. The programme also brought pleasure to the proficient readers, as they were pleasantly amazed by their ability to comprehend authentic texts.

### Rationale

The use of hypermedia and learning management systems in language education has proven to enhance student engagement and reading outcomes significantly. Research has consistently highlighted the ability of these tools to provide diverse and accessible reading materials while fostering autonomous learning experiences. Furthermore, online extensive reading programs are closely aligned with well-established Second Language Acquisition (SLA) theories, such as Krashen’s comprehensible input hypothesis and affective filter hypothesis. These theories emphasize the importance of creating opportunities for meaningful and low-anxiety learning, which digital platforms can effectively support. However, despite these advancements, there remains a gap in understanding how SLA principles can be systematically applied within the context of online extensive reading programs, particularly in settings where English is learned as a foreign language. This study aims to address these gaps by investigating the potential of adaptive digital platforms to enhance extensive reading practices through the framework of SLA. Specifically, it examines the connections between motivational, cognitive, and metacognitive factors and their combined influence on English reading comprehension in an online setting. Although prior studies have explored the roles of motivation and metacognitive strategies in reading, few have extended these findings to online extensive reading. Moreover, limited research has focused on how adaptive systems can use student performance data to personalize learning experiences, thus aligning with SLA principles like individualized learning and scaffolding. By building on existing findings, this study seeks to provide deeper insights into how technology can bridge theoretical and practical gaps in the field of SLA. It aims to offer practical recommendations for developing effective online extensive reading programs that support diverse learner needs, making a valuable contribution to both research and practice in language education.

### Hypotheses and research questions

**Fig. 1:**
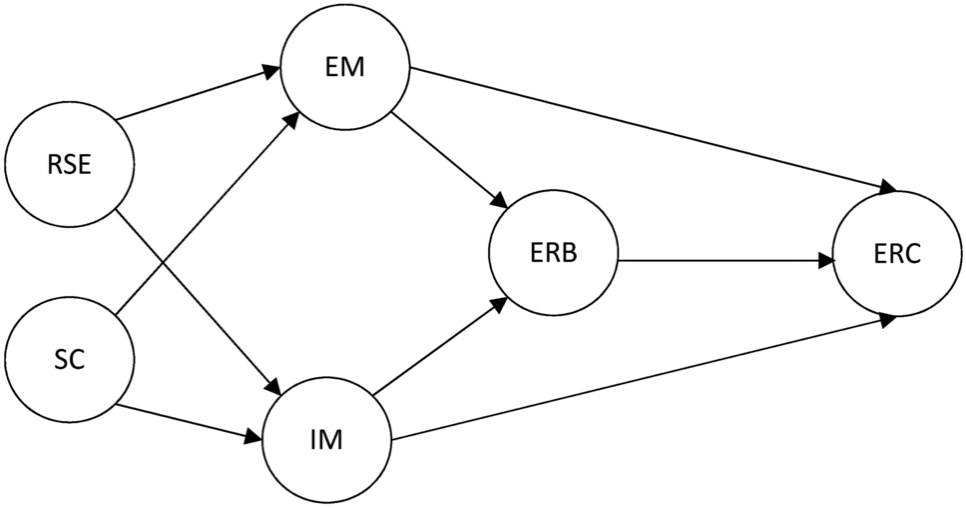
Hypothesized model of related aspects of reading. Note: EM = extrinsic motivation, ERB = English reading behavior, ERC = English reading comprehension, IM = intrinsic motivation, RSE = Reading self-efficacy, SC = self-concept.

Based on the aforementioned literature review, we proposed the following model and hypotheses as in Fig. 1

We formulated the following research questions:

- RQ1: In what types of reading comprehension questions do students find success when using an online ER program?
- RQ2: What is the effect of online ER on students’ reading comprehension?
- RQ3: What are the structural associations between affective aspects, English reading behavior, and English reading motivation?
- **H1:** Online extensive reading significantly affects students’ ability to answer vocabulary questions in a reading comprehension test^36^.
- **H2:** Students involved in compelling online ER have higher ERC achievement than those in non-compelling online ER, and students involved in non-compelling online ER have higher ERC achievement than those in offline ER activities^10,12^.
- **H3**: IM is positively associated with ERC and ERB^25,37^.
- **H4**: SC is positively associated with EM and IM^16^.
- **H5**: IM mediates the relationship between SC and ERC^2,33,37^.
- **H6**: RSE is positively associated with EM and IM^20,38^.
- **H7**: IM mediates the relationship between RSE and ERC^3,21,22^.
- **H8**: IM mediates the relationship between RSE and ERB^3,39^.
- **H9**: EM is positively associated with ERC and ERB^6^.
- **H10**: ERB mediates the relationship between IM and ERC^40,41^.
- **H11** : ERB is positively associated with ERC^27^.

## Methods

### Participants

In this study, we employed clustered randomized sampling. Initially, we estimated that there were 500 students majoring in English in Bandar Lampung, after which we identified four prominent universities that offered programs in English education. These four universities are representative of the larger population of English majors in Bandar Lampung since no other universities offer an English language education program. Then, two of the universities were randomly selected, and every fifth-semester student who had completed an intensive reading program was chosen from each of the two universities. Finally, we divided the 350 remaining students into three groups: a control group (N = 100), experimental group 1 (N = 120), and experimental group 2 (N = 130). We have two experimental groups and one control group. The total number of students involved in this study was 350 students. We divided 250 students from university A randomly into Experimetal Group 1 (120 students) and Experimental Group 2 (130 students). From university B, 100 students were assigned to the control group.

### Materials

#### Pre- and post-tests

Pre- and post-tests from the reading comprehension portion of the Test of English as a Foreign Language (TOEFL) were administered to the students. Overall, there was a total of 50 items, including six questions on identifying the main idea, 14 on understanding vocabulary in context, five on making inferences, 19 on locating specific information, and six on answering unstated detail questions. The selection of the TOEFL reading section items was due to the TOEFL validity and reliability in its use in the university context^42,43^.

#### Student questionnaires

First, the academic SC items were derived from García-Grau et al.^44^ since according to them, the items can be used in various context. Specifically, the four items highlighted the perceptions of students and their assumptions of the perceptions of teachers about their academic SC (e.g., *I am a good student; My teachers believe that I am a good student,* etc.). The items were rated on a four-point scale ranging from 1 (*very different from me*) to 4 (*a lot like me*). Based on Cronbach’s alpha of 0.97, the reliability of the items was very high.

Second, this study adopted the Motivations for Reading Questionnaire (MRQ) by Wigfield and Guthrie^45^ in order to measure RSE, IM, and EM. The MRQ, a well-established instrument for reading motivation, includes 11 constructs in three categories: competence beliefs (RSE), intrinsic and extrinsic reading motivation, and social reasons for reading. We selected five items for RSE (e.g., *I am a good reader*), 10 items for IM (e.g., *I read about my hobbies to learn more about them*), and four items for EM (e.g., *I read to improve my grades*). According to Wigfield and Guthrie^45^, the MRQ is an established instrument for reading motivation, which has been validated in various contexts.The items were rated on a four-point scale, ranging from 1 (*very different from me*) to 4 (*a lot like me*). Based on Cronbach’s alpha of 0.915, the reliability of the items was very high. We used four-point-Likert scale in our MRQ use because we followed the original scale used in the original MRQ. The original MRQ consists of eleven dimensions with a total of 50 items. They are Reading efficacy (three items), reading challenge (five items), Reading Curiosity (six items), Reading Involvement (six items), Importance of Reading (two items), Reading Work Avoidance (four items), Competition in Reading (six items), Recognition for Reading (five items), Reading for Grades (four items), Social Reasons for Reading (seven items), and Compliance (five items). After carefully studying all the dimensions, we excluded Importance of Reading, Reading Work Avoidance, Social Reasons for Reading, and Compliance since most of their items were only appropriate for primary school students based on the nature of the items. For instance, in the dimension of “Recognition for Reading”, I omitted an item that would be more suitable for primary students rather than university students—*My parents often tell me what a good job I am doing in reading*. However, there were other items suitable for university students, such as *I read to improve my grades* in “Reading for Grade”, *It is very important to me to be a good reader* in “Importance of Reading, *I feel like I make friends with people in good Books* in “Reading Involvement”, and many others. I considered these items relevant for university students. Furthermore, since the data were collected during the Covid-19 outbreak in Indonesia, and after considering time efficiency for the respondents in filling out the questionnaire, we made the MRQ more concise by using only the most relevant 19 items.

Third, the ERB items were adopted from Wang et al.^23^. Here, the three items included the amount of reading during the previous month (1 = *0 books*; 2 = *1–2 books*; 3 = *3–4 books*; 4 = *more than 5 books*), the time spent on reading (1 = *5 min.*; 2 = *15 min.*; 3 = *30 min.*; 4 = *60 min. or more*), and reading frequency (1 = *almost never*; 2 = *once a month*; 3 = *once a week*; 4 = *almost every day*). Based on Cronbach’s alpha of 0.98, the reliability of the items was very high.

#### Procedure

Overall, four steps were involved in this study. First, the study was approved by the University of Szeged Institutional Review Board (IRB) that reviewed the research proposal and it established protocols to ensure the privacy of the collected information. The informed consent was obtained from all the participants involved in this study. All methods in this study were performed in accordance with the relevant guidelines and regulations. Second, after obtaining ethical approval (and with the help of two ER lecturers), we designed an extensive ER program to examine the relationship between affective variables and reading comprehension among the sample of university students. Third, we confirmed construct validity by assessing the psychometric features of the instruments^46^. Fourth, the two ER lecturers helped the experimental groups complete the online ER intervention over a period of six weeks. Specifically, the students in the experimental groups used digital reading platforms and took pre- and post-tests, whereas the students in the control group learned reading comprehension using offline ER activities. Students read any English reading materials that they got from libraries and their teachers. The teachers let their students read the reading of their choice silently. Moreover, the former reading platform allowed the students to choose the reading materials according to their preferences, while the latter used an adaptive evaluation of students’ previous reading performance and automatically decided the text that they should read.

In the intervention groups, we used two prominent digital reading platforms for online extensive reading, readtheory.org for Experimental Group 1 and er-central.com. for Experimental Group 2. The first platform provides comprehensive features for readers and instructors. As it gives unlimited free reading access and quizes to students, the platform determines students’ reading level based on their quiz performance. It records students’ reading rate so that teachers can monitor their students’ individual reading progress, and text reading history flexibly. Since the online extensive reading intervention employed digital reading platforms, the students and the teachers could do the extensive reading activities outside the classroom hours every day. As for the second platform, teachers mainly have the same access as that of the first platform’s, but the students can decide their reading level by themselves. Students also can read brief information regarding the text they are about to read. In the experimental group 1, students selected their reading materials through an adaptive evaluation system embedded within the online reading platform. This system analyzed their prior performance, including engagement and outcomes from previous reading activities, to align reading texts with their proficiency and needs. In the experimental group 2, it also tracked reading history, engagement metrics (e.g., time spent on texts and comprehension scores), but the difference is that students’ preferences are the one that recommends materials that they will read.

On the other hand, the control group used the traditional learning method in reading comprehension. The traditional teaching for the control group is that the teacher teaches the students once a week for a six-week period in a lecturing format. Each meeting lasts for 90 min. Sometimes, the teacher also allows the students to share their reading with their peers and the teacher himself. Traditional teaching involves paper-based extensive reading. The teacher and printed books or texts are the sources of reading materials.

#### Data analysis

Using WINSTEPS Rasch software (version 3.75), we investigated the students’ parameters and the difficulty of each reading comprehension item. We then calculated item and individual reliability, while standardized mean squares and z-scores were used to evaluate validity. Moreover, using SPSS 23 software, we conducted an analysis of covariance (ANCOVA) and calculated the effect size (using partial eta squared values) of the online ER program to better assess how it can affect students’ ERC scores. Finally, using Smart PLS version 4 software, structural equation modeling was performed to determine how students’ affective traits in the online ER program can affect their ERC.

## Results

### Test results

- RQ1: In what types of reading comprehension questions do students find success when using an online ER program?

**Fig. 2:**
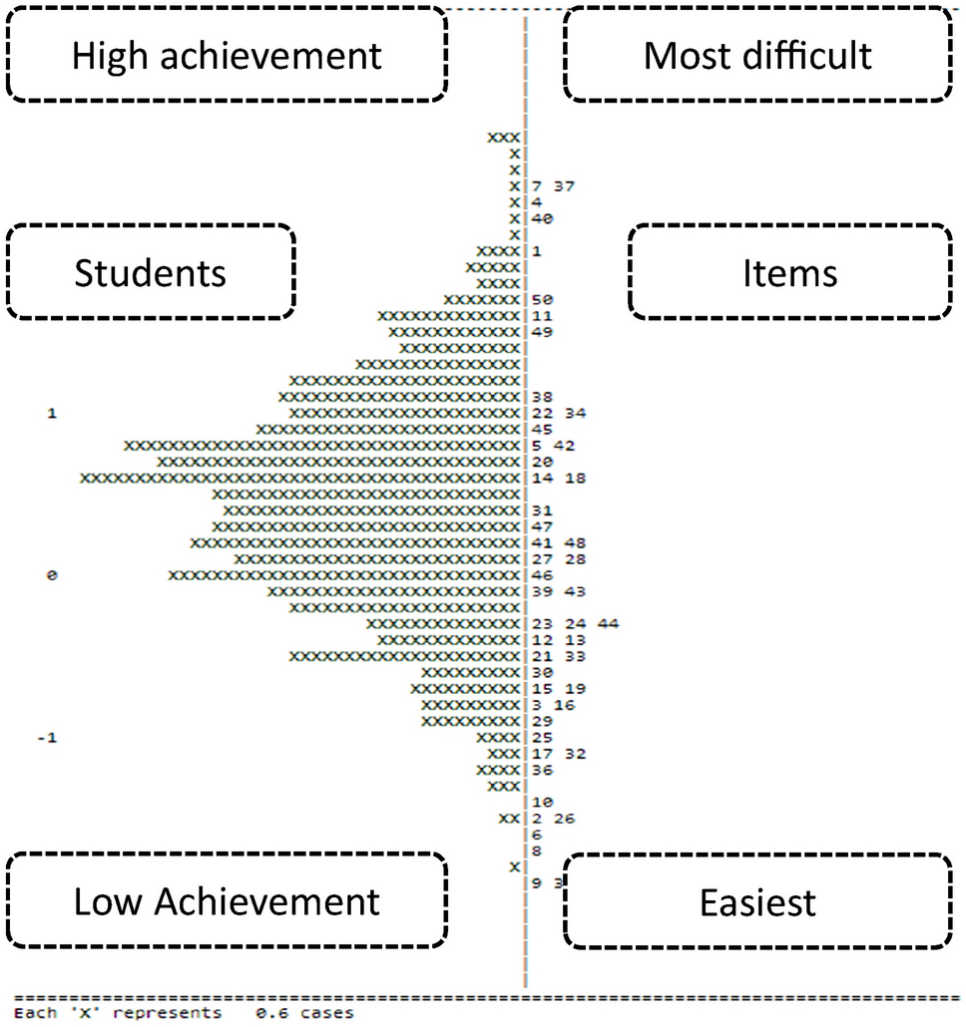
Person-item map of the students’ reading comprehension.

In this study, Rasch analysis was used to measure student achievement, compared to item difficulty. According to the person-item map in Fig. 2, which includes the students’ reading comprehension on the left and item difficulty on the right, the students performed poorly on the detail (Items 7 and 37) and inference (Item 4) questions. In this case, Item 9’s vocabulary question and Item 35’s detail question were the simplest, while the students found the other questions neither difficult nor easy.

### Reliability and fitness of the reading comprehension test

Based on the findings, the average measures (logits) for the items and individuals in experimental group 1 were 74.10 and 30.90, respectively, while in experimental group 2, the average measures for the items and individuals were 73.10 and 28.10, respectively. In the control group, the average measures for the items and individuals were 56.70 and 27.80, respectively. All of these values had positive standardized deviation values, indicating that the data collected from the sample could be used to examine the influence of online ER on students’ reading comprehension achievement^47^.

According to Table 1, the item and person reliabilities were deemed satisfactory. In addition, the internal consistency reliability scores of both the experimental and control groups were over 0.70 when using Cronbach’s alpha (KR-20). Overall, the test items in this study exhibited good reliability^48^.

**Table 1:** Reliability and fit statistics of the test for both experimental and control groups.

Construct	Experimental group 1		Experimental group 2		Control group	
	**Items**	**Persons**	**Items**	**Persons**	**Items**	**Persons**
Number	50	120	50	130	50	100
Mean	74.1	30.9	73.1	28.1	56.7	27.8
Standard deviation	22.5	8.4	27.2	7.2	35.3	5.1
Reliability (Cronbach’s alpha)	0.95	0.86	0.96	0.81	0.94	0.74
Separation	4.30	2.43	4.98	2.06	3.89	1.71
MNSQ (infit) MNSQ (outfit) ZSTD (infit) ZSTD (outfit)	0.99 1.03 -0.1 0.2	0.98 1.03 0.0 0.1	0.99 1.05 -0.2 0.2	0.99 1.05 -0.1 0.0	0.99 1.00 -0.1 0.0	1.00 0.98 -0.4 -0.1
Chi-squared (X^2^)	6174.62		6980.01		3506.78	
*df*	5831		6321		4242	

*Note. MNSQ* = *Mean Square, ZSTD* = *Z-Standard.*

This table also indicates that the mean square (MNSQ) infit/outfit values and their corresponding standardized Z-score (ZSTD) values were within the permissible range of 0.6 to 1.5 and 0.6 to 2.0, respectively^30^. Meanwhile, the chi-square values, when considering the degrees of freedom, were found to be less than 3 (± 2/df 3). Overall, the reading comprehension test was found to be suitable for evaluating the reading comprehension levels of the students.

### Analysis of covariance (ANCOVA)

- RQ2: What is the effect of online ER on students’ reading comprehension?

In this study, the ANCOVA applied student achievement (the post-test scores) as the dependent variable, the pre-test scores as covariates, and the online ER treatment (groups) as the independent variable. The homogeneity of slopes assumption in the ANCOVA was also assessed. Based on the findings, there was no statistically significant interaction (p = 0.160; p > 0.05) between the online ER treatment and the pre-test scores, indicating that this assumption was met. Moreover, after accounting for the initial differences in the pre-test scores, a statistically significant distinction was observed between the experimental and control groups in terms of their reading comprehension performance ((F (2, 349) = 6.944; p = 0.001, p < 0.001)) (see Table 2).

**Table 2:** Analysis of covariance for reading achievement (post-test) as a function of groups, using pretest scores as a covariate.

Source	df	Mean square	F-Value	*p-*Value	η2 (Eta square)
Pre-test	1	100.705	1.986	0.160	0.006
Groups	2	352.069	6.944	0.001***	0.038
Error	349	50.704			

Note: *** p < *0.001.*

Table 3 presents the mean values and standard deviations for the experimental and control groups in terms of their reading comprehension skills. According to the findings, the experimental groups, which were exposed to the online ER treatment, had superior performance in their reading comprehension, compared to the control group. The researchers also conducted an analysis of effect size, specifically partial eta squared, in order to evaluate the difference between the two groups. The results revealed a significant impact size (0.038), due to the implementation of the online ER treatment (see Table 3).

**Table 3:** Adjusted and unadjusted group means and variability for reading achievement (post-test), using pretest values as a covariate.

Group	Number	Unadjusted		Adjusted	
		**Mean**	**Standard deviation**	**Mean**	**Standard error**
Experimental 1	120	31.05	8.45	30.96	0.64
Experimental 2	130	28.12	7.18	28.07	0.62
Control	100	27.69	4.99	27.85	0.71

### Structural equation modeling

- RQ3: What are the structural associations between affective aspects, English reading behavior, and English reading motivation?

#### Descriptive statistics

Table 4 presents the descriptive statistics pertaining to the latent variables in this study. Based on the findings, all of the means exhibited values greater than 2.00 on the four-point Likert scale. This indicates that the students conveyed their active engagement, both in terms of emotional and behavioral involvement. Additionally, the observed standard deviations ranged from 0.534 to 0.815, suggesting a narrow value spread in relation to the mean outcomes.

**Table 4:** Descriptive statistics of the study construct.

Variables	Mean	SD
Reading self-efficacy	2.269	0.636
Self-concept	2.871	0.599
Intrinsic motivation	2.359	0.671
Extrinsic motivation	2.840	0.634
English reading behavior	2.019	0.534
English reading comprehension	3.019	0.815

#### Evaluation of the measurement model

Following Hair et al.^31^, a reflective measurement model was used in this study, which included evaluating the loading factors, the internal consistency of the items, convergent validity, and discriminant validity. In this regard, they recommended that the loading factor should be > 0.70. According to the loading factors in Table 5, all of the items ranged from 0.609 to 0.903. In order to examine internal reliability, composite reliability was used, instead of Cronbach’s alpha. Hair et al.^31^ also stated that a coefficient for composite reliability between 0.60 and 0.70 is considered acceptable, while that between 0.70 and 0.90 is satisfactory. In this study, the composite reliability ranged from 0.734 to 0.965. Regarding convergent validity, it refers to the extent to which the measures/constructs converge with other constructs, and appears when the cut-off value of the average variance extracted (AVE) is equal to or higher than 0.5. As shown in Table 5, the coefficient for the AVE of all the latent variables was higher than 0.5, which met these the guidelines.

**Table 5:** Convergent validity of the construct.

Latent variables	Item	Factor loading	Average variance extracted	Composite reliability
Reading self-efficacy			0.743	0.929
	SE1	0.837		
	SE2	0.875		
	SE3	0.852		
	SE4	0.876		
	SE5	0.814		
Self-concept			0.743	0.921
	SC1	0.831		
	SC2	0.854		
	SC3	0.890		
	SC4	0.873		
Intrinsic motivation			0.736	0.965
	IM1	0.895		
	IM2	0.879		
	IM3	0.903		
	IM4	0.824		
	IM5	0.799		
	IM6	0.827		
	IM7	0.883		
	IM8	0.870		
	IM9	0.827		
	IM10	0.865		
Extrinsic motivation			0.549	0.829
	EM1	0.745		
	EM2	0.787		
	EM3	0.784		
	EM4	0.637		
English reading behavior			0.500	0.734
	ERB1	0.727		
	ERB2	0.609		
	ERB3	0.737		

*Note. EM* = *extrinsic motivation, ERB* = *English reading behavior, ERC* = *English reading comprehension, IM* = *intrinsic motivation RSE* = *Reading self-efficacy, SC* = *self-concept.*

According to Fornell and Larcker^32^, assessing discriminant validity involves examining the correlation between the factors and the square root of the AVE. If this square root exceeds the correlation across these components, then it indicates the presence of potential multicollinearity^31^. According to Table 6, the AVE had a greater value, compared to the correlation between the factors. Hence, the discriminant validity of the factors were deemed adequate.

**Table 6:** Discriminant validity.

Variables	EM	ERB	IM	RSE	SC
EM	**(0.741)**				
ERB	0.192**	**(0.794)**			
IM	0.223**	0.780**	**(0.958)**		
RSE	0.289**	0.738**	0.913**	**(0.851)**	
SC	0.436**	0.130*	0.214**	0.272**	**(0.862)**

Note. *p < 0.05, ** p < *0.001, EM* = *extrinsic motivation, ERB* = *English reading behavior, ERC* = *English reading comprehension, IM* = *intrinsic motivation RSE* = *Reading self-efficacy, SC* = *self-concept.*

#### Hypothesis testing for the structural association model

**Fig. 3:**
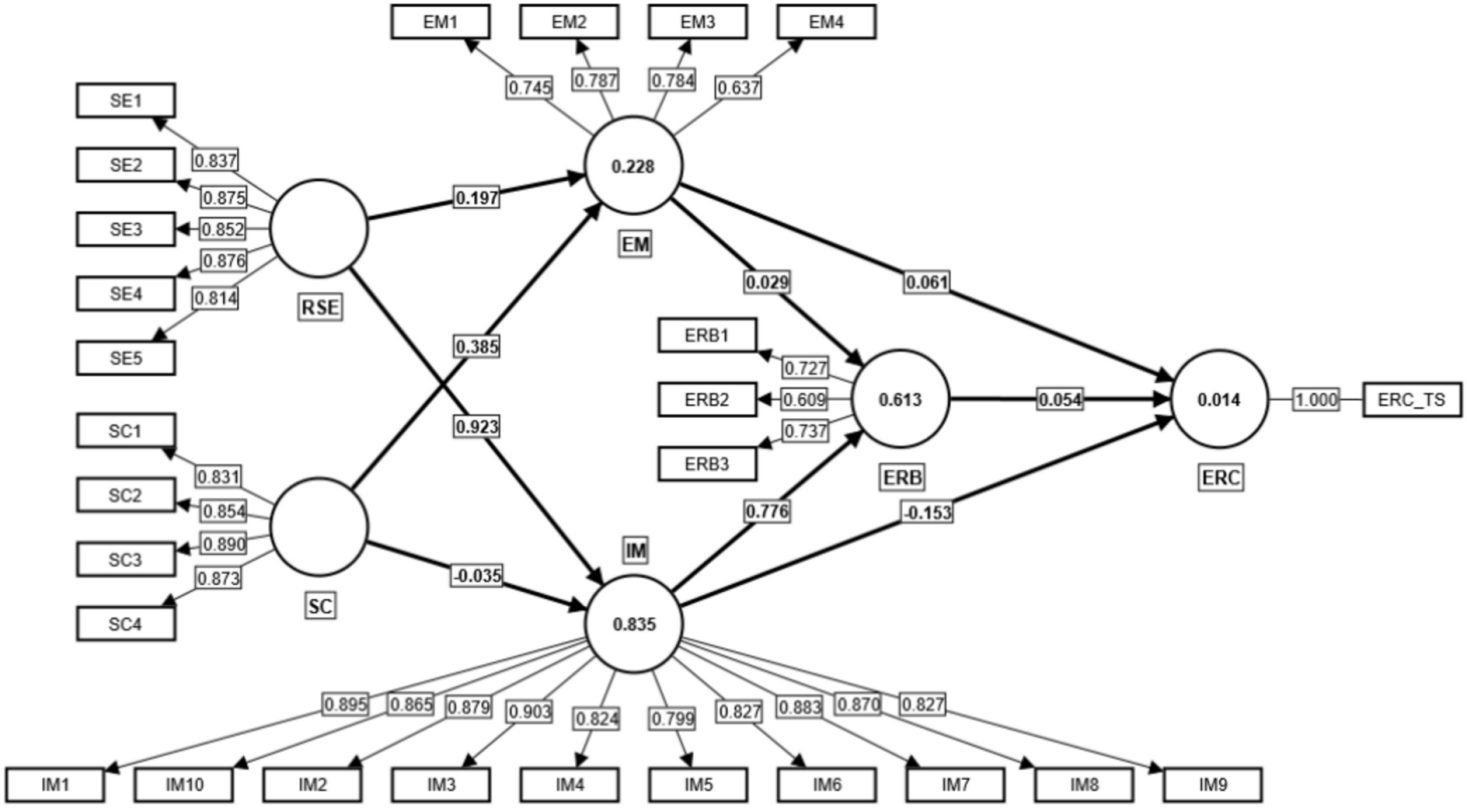
The standardized relationship between the factors. *Note: EM* = *extrinsic motivation, ERB* = *English reading behavior, ERC* = *English reading comprehension, IM* = *intrinsic motivation, RSE* = *Reading self-efficacy, SC* = *self-concept.*

In general, hypothesis testing encompasses the determination of coefficients (R^2^), the measurement of cross-validated redundancy by blindfolding (Q^2^), and the evaluation of the statistical significance/relevance of the path coefficient. We analyzed our data based on the hypothesized model as in Fig. 1. As shown in Fig. 3, the level of ERC may be accounted for by various factors (e.g., EM, IM, and ERB), which collectively explain 1.4% of the variance (R^2^ = 0.014). Specifically, the relationship between ERB and both EM and IM was accounted for 61.3% of the variance (R^2^ = 0.613).

The relationship between EM and IM was also examined in relation to RSE and SC. The results indicated that the former accounted for 22.8% (R^2^ = 0.228) of the variance in EM, while the latter accounted for 83.5% (R^2^ = 0.835) of the variance in IM. According to Hair et al.^31^, in terms of potential blindfolding-based cross-validated redundancy, it is necessary for the value of Q^2^ to exceed zero in order to demonstrate predictive accuracy for a particular endogenous construct. In this regard, the coefficients of Q^2^, ranging from 0 to 0.25 and 0 to 0.5, respectively, are indicative of low, medium, and high prediction accuracy. In the present study, the prediction accuracies of RSE and SE on EM and IM were found to be medium (Q^2^ = 0.117) and high (Q^2^ = 0.609), respectively. As for the ERB, the prediction accuracies of both EM and IM yielded a moderate effect size (Q^2^ = 0.289). In sum, the impact of EM, IM, and ERB on ERC was low (Q^2^ = 0.003).

Furthermore, this study assessed the statistical significance of the path coefficients. Figure 3 depicts the model, in which the standardized path coefficients illustrate the relationship between the different elements. Based on the findings, there was a positive relationship between RSE and EM (β = 0.197, p = 0.001) and IM (β = 0.923, p = 0.000), indicating that SC had a significant positive effect on EM (β = 0.385, p = 0.000), but no such effect on IM (β = -0.035, p = 0.190). The findings also indicate that there was no significant relationship between EM and both ERB (β = 0.029, p = 0.523) and ERC (β = 0.061, p = 0.354). However, IM had a significant positive effect on ERB (β = 0.776, p = 0.000), whereas it had no such effect on ERC (β = -0.153, p = 0.108). Meanwhile, the sole factor of ERB did not demonstrate a significant association with ERC (β = 0.054, p = 0.585). In regard to indirect prediction, only RSE had an indirect effect on ERB (β = 0.716, p = 0.000) through the mediating factor of IM. Interestingly, the remaining indirect forecasts yielded negligible results (see Table 7).

**Table 7:** The results from the hypothesis test.

Path	Estimate	t-value	p-value	Result
EM—> ERB	0.029	0.639	0.523	Not supported
EM—> ERC	0.061	0.926	0.354	Not supported
ERB—> ERC	0.054	0.546	0.585	Not supported
IM—> ERB	0.776	34.010	0.000	supported
IM—> ERC	-0.153	1.610	0.108	Not supported
RSE—> EM	0.197	3.205	0.001	supported
RSE—> IM	0.923	79.492	0.000	supported
SC—> EM	0.385	6.328	0.000	Supported
SC—> IM	-0.035	1.310	0.190	Not supported
RSE—> IM—> ERC	-0.141	1.605	0.109	Not supported
RSE—> IM—> ERB	0.716	30.189	0.000	Supported
SC—> IM—> ERC	0.005	0.945	0.345	Not supported
IM—> ERB—> ERC	0.042	0.543	0.587	Not supported

## Discussion

The first research question focused on the cognitive processes that the students found to be either easy or difficult. In this study, the students did not find such processes to be entirely easy or difficult. However, they stated that the main idea questions were more difficult, whereas the vocabulary questions were somewhat easier. This finding is in line with Chrisbianto and Aeni^39^, who found that Indonesian university students tend to have difficulty analyzing main idea questions, due to their limited vocabulary and grammatical knowledge. Thus, Indonesian EFL teachers must carefully assess text complexity before administering reading comprehension tests^49^.

The second research question investigated whether online ER can improve reading comprehension. An ANCOVA was used to account for the online ER treatment and the students’ pre-test scores when interpreting the findings. Specifically, by controlling for the pre-test scores, we isolated the effects of the online ER treatment and ruled out other factors that could explain the differences in reading comprehension achievement among the groups. The ANCOVA also showed that the online ER treatment improved the students’ reading comprehension, with the experimental groups outperforming the control group. This is in line with previous studies, which indicated that online ER includes hypermedia can help students grasp certain texts^10,12^, positive reading habits and online ER can boost students’ reading achievement^7,8,50^, and comprehensible inputs can promote language acquisition. In the present study, the students’ performances in the first experimental group were better than those in the second experimental group, even though Er-Central.com provided the students with more latitude in choosing texts than ReadTheory.org. According to Chanthap and Wasanasomsithi^40^, a good learning management system is essential for online ER. Hence, teachers/lecturers should utilize such systems in online ER to help students achieve their reading comprehension goals. Thus, this finding rejects our hypothesis that a compelling online ER group would outperform the non-compelling online ER group. However both online ER groups outperform the offline ER group.

The third research question focused on the structural relationship between affective factors, ERB, and English reading motivation. The findings are as follows. First, RSE predicted both IM and EM, which is in line with Schöber et al.^28^ and Schunk and DiBenedetto^17^, who found that self-efficacy significantly affects motivation. In related research, Yang et al.^3^ stated that students with high self-efficacy tend to be avid readers who are motivated to accomplish any reading challenge, while Carroll and Fox^41^ indicated that self-efficacy is a context-specific judgment of competence. In this regard, in order to motivate students to read, teachers should consider context-specific self-efficacy elements.

Second, SC only predicted EM, but not IM. This partially rejects the claim of Wigfield and Eccles^13^ that SC can predict both types of motivations, whereas it supports the argument of Carroll and Fox^41^ that SC can predict motivation. Here, since self-confident students tend to believe that their cognitive ability is stable, they want to prove it to their professors, family, and friends. However, regardless of the inconsistent findings of SC in predicting motivation, teachers should use it to boost their students’ reading motivation.

Third, this study revealed that neither EM nor IM predicted ERC. This was unexpected because there were numerous studies that supported this prediction^4,40,51^. According to Barber and Klauda^52^ and Machfudi and Ferdiansyah^53^, in order to have good achievement in reading, students should engage in reading activities with interest. Beglar et al. (2012)^34^ also pointed out that providing reading pleasure is a motivator and that this also affects reading performance. However, one of the reasons for our contrasting findings is the possibility that confounding variables, such as socio-economic status, cognitive ability, etc., might have rendered the prediction as more complex^54^. Although not evidenced in our results, Yang et al.^3^ argued that RSE and reading behavior have more predictive power toward reading achievement than reading motivation. Moreover, evidence of the positive association between reading motivation and reading achievement has only been found in elementary and middle school students^55^. Thus, more studies on university students’ reading motivation and achievement are required to enrich the evidence.

Fourth, the findings of this study indicated that EM did not influence ERB, whereas IM had a positive impact. In this case, the former did not support the study of Sani et al.^4^, while the latter supported the research of Marsh et al.^37^’s, Miyamoto et al.^22^’s, and Anggia et al.^5^’s. Since being intrinsically motivated in reading leads to high reading behavior, teachers should support their students’ IM prior to increasing their reading behavior. Conversely, reading behavior did not significantly predict reading comprehension, which is in contrast to Pfost et al.^24^. This is quite surprising since being an active reader generally enables one to easily comprehend an English text. Since there must be other variables that mediate reading behavior with reading comprehension, teachers should not solely rely on students’ reading behavior if they want to improve their reading achievement.

Finally, regarding the mediating effect, only IM mediated self-efficacy with reading behavior. This implies that IM is the underlying causal mechanism between self-efficacy and reading behavior, which is in line with the findings of Luther^19^ and Yang et al.^3^. Consequently, we can conclude that IM and self efficacy are two important factors in determining reading behavior, which is logical, since IM has a significant direct influence on such behavior. Conversely, the other mediation effects were insignificant. For instance, IM did not mediate self-efficacy and SC with reading comprehension, which is in contrast to numerous studies^3,3,18,19,21,22,42^_._ However, this finding was reasonable since EM and IM did not directly influence reading achievement. In this regard, it is possible that other factors, such as ER treatment and reading strategy training, have a more direct influence on reading achievement. Similarly, reading behavior did not mediate IM with reading comprehension, which is contrast to Miyamoto et al.^22^ and Wang et al.^23^, who found that reading behavior plays a significant role in mediating IM and reading achievement. Therefore, the findings of this study indicate that IM and reading behavior cannot guarantee students’ reading achievement.

### Limitations and future directions

This study includes several limitations that should be noted. First, it only modeled reading motivation by using self-determination and self-related beliefs. Thus, future research should add technology acceptance and metacognitive strategy awareness models in order to account for students’ reading motivation. Second, since this study only included English majors, students in other majors should be involved in future studies. Third, more qualitative explanations of students’ reading motivation and achievement are necessary to generalize the results. Alternatively, students’ reading comprehension could be investigated while they report aloud on the steps of the task solving. Observations could be supplemented with interviews in which students report on their reading habits and articulate their reasoning. Furthermore, qualitative analysis could shed light on learners’ text processing strategies by analysing learners’ reading diaries, where they document how they process the texts they read.

The research has highlighted the importance of examining reading and the affective factors associated with it. An important finding of the present research is the necessity of integrating authentic online resources in the teaching and learning of reading comprehension within classroom settings. However, reading is influenced by various factors, future studies could explore additional factors beyond self-efficacy, self-concept, reading behaviour and motivation. It would be beneficial to investigate the impact of reading strategies and self-regulated text processing from a reflective, metacognitive approach. Teaching should also focuse on fostering a positive attitude towards reading, which will ensure long-term reading enjoyment and pleasure beyond the classroom.

## Conclusion

This study designed an online ER program to examine the relationship between affective variables and reading comprehension among a sample of university students. The findings are as follows. First, self-related beliefs, particularly IM, influenced student reading motivation, while IM mediated the relationship between RSE and ERB. Second, reading achievement was unrelated to reading motivation and behavior, while self-efficacy affected reading behavior through IM. In this regard, reading behavior cannot associate IM and EM with reading success. Third, since reading motivation (especially IM) played a significant role in the students’ positive reading attitudes in this study, we can conclude that self-efficacy and IM are somewhat connected, providing a new goal for future research. Overall, the findings indicate that online ER can help students learn a language and develop their reading habits by providing comprehensible inputs. Therefore, in order to foster intrinsic reading motivation and improve reading performance among students, teachers should examine their self-efficacy profiles in more detail and customize motivational techniques.

## Data Availability

The datasets used and/or analyzed during the current study available from the corresponding author on reasonable request.

## References

[1] Habók, A., Magyar, A., Németh, M. B. & Csapó, B. Motivation and self-related beliefs as predictors of academic achievement in reading and mathematics: Structural equation models of longitudinal data. *Int. J. Educ. Res.* **103**, 101634 (2020).

[2] Locher, F. M., Becker, S., Schiefer, I. & Pfost, M. Mechanisms mediating the relation between reading self-concept and reading comprehension. *Eur. J. Psychol. Educ.* **36**, 1–20 (2021).

[3] Yang, G., Badri, M., Al Rashedi, A. & Almazroui, K. The role of reading motivation, self-efficacy, and home influence in students’ literacy achievement: A preliminary examination of fourth graders in Abu Dhabi. *Large-Scale Assess. Educ.*10.1186/s40536-018-0063-0 (2018).

[4] Sani, A. M., Ariffin, T. F. T. & Shaik-Abdullah, S. I’ll read in english if...: A glimpse into the nature of tertiary ESL reading motivation. *Proced. - Soc. Behav. Sci.* **118**, 343–350 (2014).

[5] Schöber, C., Schütte, K., Köller, O., McElvany, N. & Gebauer, M. M. Reciprocal effects between self-efficacy and achievement in mathematics and reading. *Learn. Individ. Differ.* **63**, 1–11 (2018).

[6] Mikami, A. students’ attitudes toward extensive reading in the Japanese EFL context. *TESOL J.* **8**, 471–488 (2017).

[7] Elturki, E. & Harmon, E. Systematic integration of extensive reading in the curriculum: Strategies and resources. *TESOL J.* **11**, 1–12 (2020).

[8] Stoller, F. L. & Nguyen, L. T. H. Reading habits of Vietnamese University English majors. *J. English Acad. Purp.* **48**, 100906 (2020).

[9] Patrick, R. Comprehensible input and Krashen’s theory. *J. Class. Teach.* **20**, 37–44 (2019).

[10] Krashen, S. D. In *Principles and practice in second language acquisition / Stephen D. Krashen.* (1982)

[11] Chavangklang, T., Chavangklang, P., Thiamhuanok, S. & Sathitdetkunchorn, P. Development of EFL university students’ vocabulary size and reading comprehension using online multimedia-based extensive reading. *Adv. Lang. Lit. Stud.* **10**, 146 (2019).

[12] Komala, A. S. & Rifai, I. The impacts of the cherry orchard video game on players’ reading comprehension. *Proced. Comput. Sci.* **179**, 368–374 (2021).

[13] Shepard-Carey, L. Making sense of comprehension practices and pedagogies in multimodal ways: A second-grade emergent bilingual’s sensemaking during small-group reading. *Linguist. Educ.* **55**, 100777 (2020).

[14] Singer Trakhman, L. M., Alexander, P. A. & Silverman, A. B. Profiling reading in print and digital mediums. *Learn. Instr.* **57**, 5–17 (2018).

[15] Yum, Y. N., Cohn, N. & Lau, W. K. W. Effects of picture-word integration on reading visual narratives in L1 and L2. *Learn. Instr.* **71**, 101397 (2021).

[16] Rezaee, M. & Farahian, M. Promoting university students’ receptive skills through extensive reading in multimedia-based instruction. *J. Appl. Res. High. Educ.*10.1108/JARHE-09-2020-0304 (2020).

[17] Chanthap, N. & Wasanasomsithi, P. The effect of integration of a blended learning and extensive reading instructional model on Thai efl undergraduate students’ learner autonomy. *Learn. J. Lang. Educ. Acquis. Res. Netw.* **12**, 76–96 (2019).

[18] Cote, T. & Milliner, B. Extensive Reading on Mobile Devices : Is it a worthwhile strategy? *Proc. 12th Asia TEFL 23rd MELTA Int. Conf.* 979–990 10.13140/2.1.3812.2885 (2014).

[19] Milliner, B. & Cote, T. One year of extensive reading on mobile devices: Engagement and impressions. In *Critical CALL – Proceedings of the 2015 EUROCALL Conference, Padova, Italy*, (eds Helm, F. et al.) 404–409 (Dublin: Research-publishing.net., 2015). 10.14705/rpnet.2015.000366

[20] Shurentsetseg, Nandintsesteg & Nyamsuren, A. Implementing Extensive Reading through Xreading.com for Mongolian EFL Learners. In *Proc. of the fifth world congress on extensive reading* (2015).

[21] Keller, J. M. First principles of motivation to learn and e3-learning. *Distance Educ.* **29**, 175–185 (2008).

[22] Al Zeidi, J. & Al Quraini, B. Moodle as an extensive reading mechanism: A study to facilitate extensive reading in an omani Efl context. *PEOPLE Int. J. Soc. Sci.* **5**, 350–365 (2019).

[23] Cheetham, C., Harper, A., Elliott, M. & Ito, M. Assessing student attitudes toward graded readers, mreader and the mreader challenge. *Read. Matrix An. Int. Online J.* **16**, 1–19 (2016).

[24] HAGLEY, E. T. Extensive graded reading with engineering students : Effects and outcomes. *Read. A. Foreign Lang.* **29**, 203–217 (2017).

[25] Matsuda, S. Sharing reading experiences with university students using goodreads. *J. Extensive Read.* **5**, 77–86 (2020).

[26] Suk, N. L2 Students’ perceptions of MReader for extensive reading. *Multimed. –Assist. Lang. Learn.* **21**, 163–180 (2018).

[27] Pfost, M., Dörfler, T. & Artelt, C. Students’ extracurricular reading behavior and the development of vocabulary and reading comprehension. *Learn. Individ. Differ.* **26**, 89–102 (2013).

[28] Locher, F. M., Becker, S. & Pfost, M. The relation between students’ intrinsic reading motivation and book reading in recreational and school contexts. *AERA Open* **5**, 1–14 (2019).

[29] Guay, F., Stupnisky, R., Boivin, M., Japel, C. & Dionne, G. Teachers’ relatedness with students as a predictor of students’ intrinsic motivation, self-concept, and reading achievement. *Early Child. Res. Q.* **48**, 215–225 (2019).

[30] Park, Y. How motivational constructs interact to predict elementary students’ reading performance: Examples from attitudes and self-concept in reading. *Learn. Individ. Differ.* **21**, 347–358 (2011).

[31] Sutter, C. C. & Campbell, L. O. The role of academic self-determined reading motivation, reading self-concept, home reading environment, and student reading behavior in reading achievement among American Indian and Hispanic students. *Contemp. Educ. Psychol.* **70**, 102093 (2022).

[32] Katzir, T., Lesaux, N. K. & Kim, Y. S. The role of reading self-concept and home literacy practices in fourth grade reading comprehension. *Read. Writ.* **22**, 261–276 (2009).

[33] Paumier, D. & Chanal, J. The differentiated mediation effect of academic autonomous and controlled motivation in the relation between self-concept and achievement. *Learn. Motiv.* **83**, 101918 (2023).

[34] Beglar, D., Hunt, A. & Kite, Y. The effect of pleasure reading on Japanese university EFL learners’ reading rates. *Lang. Learn.* **62**(3), 665–703 (2012).

[35] Arnold, N. Online extensive reading for advanced foreign language learners: An evaluation study. *Foreign Lang. Ann.* **42**(2), 340–366 (2009).

[36] Arnold, N. Online extensive reading for advanced foreign language learners: An evaluation study. *Foreign Lang. Ann.* **42**, 340–366 (2009).

[37] Huang, C. Self-concept and academic achievement: A meta-analysis of longitudinal relations. *J. Sch. Psychol.* **49**, 505–528 (2011). 21930007 10.1016/j.jsp.2011.07.001

[38] Schunk, D, H. & DiBenedetto, M, K. Self-efficacy theory in education. In *Handb. motiv. Sch. *2nd edn 10.4324/9781315773384 (2016)

[39] Luther, V. L. The impacts of self-efficacy and intrinsic motivation: Mentoring students to be motivated readers. *Lang. Lit. Spectr.* **32** (2022).

[40] Miyamoto, A., Pfost, M. & Artelt, C. The relationship between intrinsic motivation and reading comprehension : Mediating effects of reading amount and metacognitive knowledge of strategy use. *Sci. Stud. Read.*10.1080/10888438.2019.1602836 (2019).

[41] Wang, X., Jia, L. & Jin, Y. Reading amount and reading strategy as mediators of the effects of intrinsic and extrinsic reading motivation on reading achievement. *Front. Psychol.* **11**, 1–16 (2020). 33192919 10.3389/fpsyg.2020.586346 PMC7652739

[42] Vu, L. T. & Vu, P. H. Is the TOEFL score a reliable indicator of international graduate students’ academic achievement in american higher education? executive manager of e-center for professional development phd (abd) in curriculum and instruction. *Int. J. Stud. English Lang. Lit.* **1**, 11–19 (2013).

[43] Pranoto, Y. H. The effect of the TOEFL preparation program on reading skills and structure mastery of prospective students. *Jet. Adi. Buana.* **5**, 77–88 (2020).

[44] García-Grau, P., Ayora Pérez, D., Calabuig Moreno, F. & Prado-Gascó, V. J. Self-concept in preadolescence: A brief version of AF5 scale. *Motriz. Rev. Educ. Fis.* **20**, 151–157 (2014).

[45] Wigfield, A. & Guthrie, J. T. Relations of children’s motivation for reading to the amount and breadth of their reading. *J. Educ. Psychol.* **89**, 420–432 (1997).

[46] Rejeki, S. et al. Discrimination index, difficulty index, and distractor efficiency in MCQs English for academic purposes midterm test. *J. English Lang. Pedagog.* **6**, 1–11 (2023).

[47] Polat, M., Turhan, N. S. & Toraman, Ç. Comparison of classical test theory vs. multi-facet rasch theory. *Pegem Egit. ve Ogr. Derg.* **12**, 213–225 (2022).

[48] Ismail, N. E., Jimam, N. S., Dapar, M. L. P. & Ahmad, S. Validation and reliability of healthcare workers’ knowledge, attitude, and practice instrument for uncomplicated malaria by rasch measurement model. *Front. Pharmacol.* **10**, 1–8 (2020). 10.3389/fphar.2019.01521 PMC6968786 31998125

[49] Anggia, H. & Habók, A. Textual complexity adjustments to the English reading comprehension test for undergraduate EFL students. *Heliyon*10.1016/j.heliyon.2023.e12891 (2023). 36699275 10.1016/j.heliyon.2023.e12891 PMC9868442

[50] Hwang, H. B., Coss, M. D., Loewen, S. & Tagarelli, K. M. Acceptance and engagement patterns of mobile-assisted language learning among non-conventional adult L2 learners: A survival analysis. *Stud. Sec. Lang. Acq.*10.1017/S0272263124000354 (2024).

[51] Marsh, H. W., Köller, O., Trautwein, U., Lüdtke, O. & Baumert, J. Academic self-concept, interest, grades, and standardized test scores: Reciprocal effects models of causal ordering. *Child Dev.* **76**, 397–416 (2005). 15784090 10.1111/j.1467-8624.2005.00853.x

[52] Smadi, O. & Al-Zawahreh, A. The effect of an online extensive reading instructional program on jordanian eleventh grade students. *Profic. English* **4**, 170–188 (2013).

[53] Machfudi, M. I. & Ferdiansyah, S. A tale from extensive readers in an online extensive reading classroom. *Qualit. Res. J.* **23**(4), 420–426 (2023).

[54] Zhang, J. *et al.* Reading motivation, reading achievement, and reading achievement gaps: Evidence from the NAEP 2015 reading assessment AIR-NAEP working paper 2020–01 *Am. Institutes Res* 1–39 (2020).

[55] Wang, X. & Jin, Y. A Validation of the Chinese motivation for reading questionnaire. *J. Lit. Res.* **53**, 336–360 (2021).

